# Addison's Disease Revisited in Poland: Year 2008 versus Year 1990

**DOI:** 10.4061/2010/731834

**Published:** 2010-06-06

**Authors:** Anna A. Kasperlik-Zaluska, Barbara Czarnocka, Wojciech Jeske, Lucyna Papierska

**Affiliations:** ^1^Department of Endocrinology, Centre for Postgraduate Medical Education, Bielanski Hospital, Ceglowska 80, 01-809 Warsaw, Poland; ^2^Department of Biochemistry and Molecular Biology, Centre for Postgraduate Medical Education, Bielanski Hospital, Ceglowska 80, 01-809 Warsaw, Poland

## Abstract

This study aimed at comparing two groups of patients with Addison's disease: A, including 180 patients described in 1991 and B, consisting of 138 patients registered since 1991. The incidence of coexisting autoimmune disorders was evaluated and etiological factors were analyzed. Immunological and imaging studies (computed tomography in group B) were performed. Adrenal autoantibodies were examined by an indirect immunofluorescence technique in group A, and by the assay measuring autoantibodies against steroid 21-hydroxylase in group B. Adrenal autoantibodies were revealed in 37% of patients examined by the immunofluorescence method and in 63% investigated by the modern technique. Tuberculosis was found in 52 patients in the group A and in two patients in the group B; metastatic infiltrations of the adrenals in CT were detected in 16 patients. Probable autoimmune Addison's disease was diagnosed in 125/180 patients (69%) in the group A and in 116/138 patients (84%) in the group B.

## 1. Introduction

For many years tuberculosis has been considered as the main cause of primary adrenal insufficiency [1]; however, new therapeutic methods and recent diagnostic procedures changed our view on its etiology [2]. We have been collecting and studying patients with adrenal insufficiency for over 40 years and we would like to present our experience in this matter. Our present material consists of 318 patients with Addison's disease (AD) and 301 patients with the so-called idiopathic isolated secondary adrenal insufficiency (a part of this later group was described in 1998 [3] and in 2003 [4]). In 1991 we published a report on 180 patients with AD, observed for 1 to 26 years [5]. From this time 138 new patients were referred to our department and observed for 1 to 18 years. The aim of this study was to compare incidence of autoimmune disorders and frequency of tuberculosis as well as metastatic destruction of the adrenals in these two groups of patients with AD, using more modern techniques of diagnosis in the group observed from 1990.

## 2. Materials and Methods

### 2.1. Patients

180 patients with AD, 113 women and 67 men, aged 9 to 74 years, described in 1991 (group A) and 138 patients, 97 women and 41 men, 12 to 80 years old, registered since 1991 (group B). The patients were derived mainly from Warsaw and a neighbouring area but a significant part of them was referred to our department from other, distant clinical centers. AD was diagnosed on the basis of clinical data [6] and the results of hormonal investigations. The most prominent clinical manifestations included reduced strength, fatigability, anorexia, weight loss, nausea, skin hyperpigmentation, and postural hypotension. Hyperkalaemia and hyponatraemia were frequent findings, more severe in stress situations, presenting with myalgia, joint pain, vomiting, diarrhea, and low blood pressure. The diagnostic hormonal criteria for including the patients into the groups under study were low 17-hydroxycorticosteroids (17-OHCS) urinary excretion (below 6.0 *μ*mol/24 hours, normal: 6.1–19.3 *μ*mol), low serum cortisol levels at 09.00 hours (below 209 nmol/L, normal: 209–692 nmol/L), high plasma morning ACTH concentration (more than 13.2 pmol/L, in most cases over 50 pmol/L, normal: 3.3–13.2 pmol/L), and lack of significant increase of cortisol and/or 17-OHCS in corticotropin (Synacthen) stimulation test [5]. In two patients with autoimmune origin of AD and six patients with metastatic adrenal infiltrations or lymphoma, elevated ACTH levels, 18.4 to 42.4 pmol/L were the first sign of subclinical adrenal insufficiency.

Informed consent for the studies was obtained from all the patients.

### 2.2. Methods

In clinical examination we searched for symptoms and signs of adrenal insufficiency; every patient was also investigated for other autoimmune disorders. The probable causes of AD were analyzed. The immunological studies, performed in all patients with probable AAD, included thyroid autoantibodies determinations: thyroglobulin (aTg) and microsomal (aMic) antibodies assayed by haemagglutination tests (normal, <1/120) and antiperoxidase antibodies (aTPO) by “in house” ELISA, as described previously [7] (normal <1/80). A search for adrenal autoantibodies was performed in 52 patients in the group A by means of an indirect immunofluorescent technique [8] while in the group B a search for antibodies against steroid 21-hydroxylase (21-OH, normal <1) and aromatic L-amino acid decarboxylase (AADC, cut off =7) in 62 patients, as well as against steroid 17-hydroxylase (17-OH, cut off =11) and side-chain cleavage enzyme (SCC, cut off =13) in 9 patients with associated premature ovarian failure (POF), was conducted by the method based on the in vitro transcribed and translated protein as described by Ekwall et al. [9]. The upper normal limit of each antibody index was established as the mean value of negative controls plus three standard deviations and values above this cut-off indicated the presence of adrenal autoantibodies. Additional hormonal measurements: thyroxine, T_4_ or free thyroxine, fT_4_, and TSH levels were measured initially by radioimmune assays (RIA/IRMA) and during the last 10 years by chemiluminescent assay (LIA). Serum estradiol and FSH were determined, if necessary, initially by RIA and during last 10 years by LIA. In selected patients parathormone, PTH was measured by IRMA. Various biochemical analyses were performed, if necessary, that is, serum glucose and HbA1c levels, calcium and phosphorus, serum and urinary values, and alkaline phosphatase activity. Computed tomography (CT) of the adrenals was performed in some patients from the group B.

## 3. Results

Basing on the results of the clinical examination with biochemical determinations, as well as immunological and imaging investigation, we diagnosed in the group A 125 patients (87 women, F/M ratio 2.3) with probable autoimmune etiology (69%) and 52 (26 women, F/M ratio 1.0) with a history of tuberculosis (29%). In two patients metastatic infiltrations and in one man amyloidosis of the adrenals were diagnosed at autopsy. Autoimmune disorders were found in 80 patients in the group A (64% out of 125 patients with probable autoimmune origin of AD and 44% in all the group under study). In the group B there were 105 patients (88 women, F/M ratio 5.0) with AD associated with some autoimmune disorders (76% in all the group under study), two men with tuberculosis, 16 ones (3 women, F/M ratio 0.2) with metastatic infiltrations of both adrenals (12%), four with bilateral lymphoma (two women), a 53-years-old man with bilateral adrenal cancer and 11 patients (5 women) with idiopathic AD (8%), without any abnormalities in CT (probably also of autoimmune origin). Characteristics of group A and B patients are summarized in Table 1. 

The imaging pattern of CT in other examined patients, with an early stage of AAD was normal, while in two patients with tuberculous infection adrenals were enlarged. In the patients with AAD of long-term duration atrophic adrenals were characteristic in the abdominal CT.

Autoimmune diseases coexisting with AD are summarized in Table 2 (the % values are calculated for the patients with probable autoimmune AD). Thyroid abnormalities, especially hypothyroidism and thyrotoxicosis due to Graves' disease or Hashitoxicosis, as well as insulin dependent diabetes mellitus (T1DM) were dominant autoimmune disorders. In all patients with pernicious anemia atrophic gastritis (verified by histological examinations) was found, and in majority of them it was an early clinical finding characteristic of this disease. Additionally, there were two patients with chronic active hepatitis in the group A, as well as one patient with rheumatoid arthritis, one patient with a collagen disease and one patient with Dühring's disease in the group B. In two patients secondary adrenal insufficiency appeared after three and 18 years of AD duration with loss of hyperpigmentation and a decrease of ACTH concentration from 165.6 to 4.0 and from 74 to 2.2 pmol/L, respectively. Hypoparathyroidism, diagnosed as first autoimmune disorder in five patients, was associated with moniliasis. In contrast, in a young male addisonian patient (brother of a female patient with AD, T1DM, and pernicious anemia) only moniliasis was present. Multiple autoimmune disorders were observed in 20 patients in the group A (16%) and in 19 patients (17%) in the group B.

Thyroid autoantibodies were detected in 55% of the patients tested in the group A and in 65% of the patients in group B, (aMic—in 39%, aTg—in 31%, both ranging from 1/120 to 1/6480 and aTPO in 65%, ranging from 1/80 to 1/51200).

Indirect immunofluorescent method revealed adrenal autoantibodies in 19 out of 52 patients with probable autoimmune AD (37%) in the group A. Antibodies against 21-hydroxylase were detected in 39/62 patients (63%), ranging from 2 to >5000, most frequently in the patients with recently diagnosed AD (in 17 patients it exceeded 20). Antibodies against 17-hydroxylase were found in 2/9 women with coexisting premature ovarian failure, ranging 107 and 115. Anti-SCC antibodies were revealed in the same group in five patients (56%), ranging from 19 to 82. A significant level of anti-ACDD antibodies was not found in this group of patients.

A significant level of anti-AADC antibodies was not found the mortality in the group A was mainly due to the age-related diseases and the insufficiently treated tuberculosis. Four patients died because of malignant diseases: colonic cancer, ovarian cancer, uterine cancer and myeloma. In the group B, majority of patients with metastatic destruction of adrenals as well as patients with bilateral lymphoma died due to malignancy. Nearly all our patients with AAD have been engaged in professional activities or housekeeping and they have been going well at routine control examinations. To the best of our knowledge, three of them died due to adrenal crisis (insufficient replacement therapy in stress situation), intestinal perforation, and septic shock.

The routine hydrocortisone doses ranged between 25 and 30 mg daily, with extra doses if necessary, similarly as in Erichsen et al. studies [10]. In the male patients with 180 cm of height or more, a daily dose of 40 to 45 mg was necessary. The most frequent doses of fludrocortisone ranged from 0.05 mg to 0.1 mg in the morning, however in some patients only 0.025 mg has been a tolerable dosing, and individual patients withheld this drug because of oedema. In some patients 60 years old or older, we had to withdraw fludrocortisone because of the blood pressure increase induced by this drug.

## 4. Discussion

In this paper we present a group of 318 patients with Addison's disease registered at our department in the last 48 years. The group of patients observed till 1990 (group A) compared to the group registered in the last 18 years (group B) presented a different etiologic profile. Effective chemotherapy of tuberculosis significantly decreased the number of patients with this type of adrenal destruction, in our material from 29% to 1.5%. At present, only in the developing countries tuberculous adrenalitis remains a major etiologic factor [11]. In contrast, detectability of metastatic infiltrations of adrenal glands increased due to modern imaging techniques from 1% in the group A to 12% in the group B (together with bilateral adrenal lymphoma and bilateral adrenal cancer: 15%). In some cases of this later group we detected a subclinical AD with subtle symptoms and elevated plasma ACTH levels. In the course of further observation, clinical picture of AD developed in the patients whose tumors were inoperable. 

Autoimmune disorders accompanying AD were detected in 80 patients in the group A (64% of 125 patients with idiopathic AD) and in 105 patients in the group B (Table 1). The remaining 11 patients registered recently in the group B, without any evident autoimmune disorders, according to Betterle et al. [2], could be classified also as idiopathic AD of probable autoimmune etiology. Similar observations were reported by other authors [12, 13]. Thus the incidence of autoimmune disorders in the group B could be estimated as 90% in 116 patients with probable autoimmune AD and 76% in all the patients in this group. Long-term observation of such a significant group of patients with AD revealed a tendency to development of some other autoimmune disorders (mainly thyroid autoimmunity and diabetes mellitus) in the later phases of the disease, as a sign of a progressive autoimmune process.

Our material of nonautoimmune AD in the group B did not differ significantly from the observations of Falorni et al. [14]. In contrast, in the Norwegian Registry the primary adrenal insufficiency was considered as almost exclusively autoimmune disease [10].

Conventional method of indirect immunofluorescence revealed adrenal autoantibodies in 37% out of 52 patients in the group A. Modern assays of adrenal autoantibodies detected autoimmunity in 43 out of 67 patients under study (64%). This result is similar to that in Winqvist et al. study [15].

It was characteristic that the presence of adrenal autoantibodies was detected mainly in the patients with recently diagnosed AD, while in the patients with long duration of disease, adrenal antibodies were hardly detected or absent. In our opinion lack of adrenal antigen due to a complete adrenal destruction in the long-term disease could be responsible for this finding [5]. A similar suggestion was presented by Falorni et al. [14]. It must be said however that not all our patients were characterized for adrenal autoantibodies, therefore we have to be cautious in opinion on that matter.

In previous studies we found high prevalence of thyroid autoimmunity in idiopathic AD [7], which is characteristic for autoimmune polyglandular syndrome type 2 (APS 2) [2]. In the natural history of the thyroid autoimmune disease the thyrotoxic patients developed hypothyroidism during long-term observation. Thus hypothyroidism was the most frequent, clinically overt autoimmune disease associated with AD. More than a half of the patients in the group B could be classified as having Hashimoto's disease, with presence of thyroid autoantibodies (the most frequently aTPO.). Our observations suggest an increasing tendency to thyroid autoimmunity development in AAD [16]. No significant reactivity was found against AADC though it has been shown to correlate to vitiligo [17] which was rather frequent in our patients. Antibodies against AADC were found in a significant number of Scandinavian patients [18, 19].

Diabetes mellitus and pernicious anemia were more frequent in the group B, while premature ovarian failure (POF) was a slightly less frequent disorder. Several forms of POF are not of autoimmune etiology, however in a majority of our patients with premature menopause other autoimmune disorders have been observed. The patients with hypoparathyroidism as the first sign of autoimmune disease should be classified as APS1, with different genetic background as compared with our other patients from the APS2 group. 

Interestingly, we observed a conversion of two patients with AD to ACTH deficiency, probably due to development of pituitary autoimmunity.

In majority of our patients the daily dose of hydrocortisone, adjusted to their height and/or weight ranged 25–30 mg (divided in two or three doses). We are aware that such dosage exceeds slightly the normal cortisol secretion rate, but patients feel better on this dose and one cannot exclude a possibility that such a bit higher dose could delay development or alleviate the course of other autoimmune disorders. Less frequently, a dose of 20 mg of hydrocortisone was prescribed, mainly in the patients with obesity or genetically prone to obesity. Some clinical observations suggest that patients being on 15–20 mg of hydrocortisone daily do not feel fully comfortable. We cannot exclude a possibility that they present a hydrocortisone deficiency in some “substress” situations, not included in a routine stress definition. May be the oral hydrocortisone modified release tablets, mimicking the physiological serum cortisol profile, will be a better therapeutic offer for many AD patients [20].

A stable incidence of AD is suggested in the literature. In our observations however, it is the secondary adrenal insufficiency which became a more frequent adrenal disease, mainly due to the improved detectability.

## 5. Conclusions

(1) At present, in our country, tuberculosis does not play any significant role in etiology of AD. (2) Detection of metastatic lesions of adrenal glands increased evidently due to new imaging techniques. (3) Modern methods of adrenal antibodies determinations detected adrenal autoimmunity in a higher number of patients with AD than indirect immunofluorescence, more frequently in patients with AD of short duration. (4) Thyroid autoimmunity was the most frequent autoimmune disorder accompanying AD.

##  Conflict of Interest Statement

The authors declare that no financial or other relationships exist that might lead to a conflict of interest.

## Figures and Tables

**Table 1:** Number of patients, gender and age at onset of AD patients in the group A and B.

Etiology	Gender		Age at onset—years	
	F	M	median	Age borders
Group A				
Probable AAD	87	38	33	13–38
Tuberculosis	26	26	43	13–67
Neoplasia	0	2		48, 62
Amyloidosis	0	1		58

Group B				
Etiology				
Probable AAD	93	23	27	8-50
Tuberculosis	0	2		57, 78
Neoplasia	5	16	65	5–76

**Table 2:** Autoimmune diseases coexisting with Addison's disease in the group A and group B (including only the patients with probable autoimmune disease).

	Group A—total (%)		Group B—total (%)	
Hypothyroidism	17	(14)	47	(41)
Subclinical	6	(5)	5	(3)
Thyrotoxicosis	15	(12)	12	(10)
Vitiligo	17	(14)	12	(10)
POF	18	(21)*	8	(9)*
T1DM	7	(6)	17	(15)
Pernicious anemia	4	(3)	9	(8)
Alopecia areata	3	(2)	2	(2)
Hypoparathyroidism	2	(2)	3	(3)

*Calculated for women only.
